# Perinatal care for women with refugee backgrounds from African countries: a qualitative study of intersections with psychological wellbeing

**DOI:** 10.1186/s12884-022-04957-9

**Published:** 2022-08-08

**Authors:** Clemence Due, Moira Walsh, Isadora Aldam, Amelia Winter, Susan Cooper, Josephine Sheriff, Anna Ziersch

**Affiliations:** 1 The School of Psychology, The University of Adelaide, Adelaide, SA Australia; 2 College of Medicine and Public Health, Flinders University, Adelaide, SA Australia; 3 Flinders Medical Centre, Adelaide, SA Australia

**Keywords:** Refugees, Perinatal care, Africa, Wellbeing, Maternity, Pregnancy

## Abstract

**Background:**

Experiences with healthcare services, including perinatal healthcare services, contribute to psychological wellbeing for refugees post-resettlement. To address the paucity of literature examining the relationship between perinatal healthcare and psychological wellbeing in women with refugee backgrounds from African countries this study aimed to: (1) understand the relationship between psychological wellbeing and perinatal care amongst this population, and; (2) identify areas for improved perinatal healthcare services to ensure positive wellbeing outcomes in this population.

**Methods:**

A total of 39 participants were included in the study. Nineteen women from seven African countries participated in interviews – seven both prior to and after having their babies, two only while pregnant and ten only after their baby had been born. In addition, interviews were conducted with 20 service providers. Interviews were thematically analysed.

**Results:**

Four key themes were identified, covering continuity of care, cultural safety of care, agency in decision making, and ongoing impacts of perinatal care experiences.

**Conclusions:**

The results highlighted the need for changes to perinatal healthcare provision at the systems level, including implementing a continuity of care model, and ensuring women’s access to individualised, trauma-informed perinatal services which attend to the cultural and psychosocial resettlement needs of this population. These findings informed recommendations for improving perinatal healthcare services and better psychological outcomes – and in turn broader health outcomes – for African-background refugee mothers.

## Introduction

It is well established that people with refugee backgrounds living in resettlement countries such as Australia are at greater risk of poor health outcomes – including mental ill-health – compared to the general population [1, 2]. Moreover, their use of healthcare services post-resettlement is affected by various individual and systematic factors; often contributing further to mental health disparities [3, 4]. Perinatal healthcare is one such service essential for many refugee women in Australia, around 30% of whom are of child-bearing age [5]. Whilst refugee status is a risk factor for perinatal mental ill-health (and additional obstetric and other complications), challenges persist in responding to the needs of this population [6]. To address the paucity of literature examining the relationship between perinatal healthcare and psychological wellbeing in refugee women – specifically those from the African continent—this study aimed to: (1) understand the relationship between psychological wellbeing and perinatal care amongst this population, and; (2) identify potential improvements to perinatal healthcare services to promote positive psychological outcomes.

### Terminology

#### Refugee

The term ‘refugee’ refers to peoples who, due to well-founded fear of persecution, are declared unable to seek protection within their country of origin [7]. In Australia, refugees are typically granted permanent protection following referral to the Australian Government for resettlement by the United Nations High Commissioner for Refugees. Whilst this paper refers to participants as “refugees” for brevity, it is important to acknowledge that identity is complex, and refugee status only one component.

#### Psychological wellbeing

The complexities of human health – including physical, psychological and social elements – can be conceptualised holistically by the term ‘wellbeing’. Exploration of wellbeing is most appropriate in this context as it also acknowledges protective factors and subjectivity in perceptions of health status [8]. As such, the term “psychological wellbeing” or simply “wellbeing” are used in this paper to refer to both mental health and more holistic aspects such as social inclusion.

### Background and previous literature

Numerous reviews examining post-resettlement refugee wellbeing have reported Posttraumatic Stress Disorder (PTSD), depression and anxiety as highly prevalent [9, 10]. PTSD has attracted the most research globally, with refugees resettled in ‘Western’ countries at up to a ten-fold greater risk than general populations [11]. However, focusing solely on clinical symptomology and diagnosable mental health conditions arguably labels mental ill-health as an inevitable outcome of the refugee experience. On the other hand, examining psychological wellbeing offers a more holistic approach, involving the subjective evaluation of both health and potential protective factors [8]. Research has shown that wellbeing amongst refugees is impacted by post-migration challenges including gender role changes, language proficiency, unemployment, and social isolation, all of which are also mediated by individual factors such as resilience and systematic factors such as appropriate support services [12, 13].

Importantly for this paper, experiences with health care services in resettlement countries also affects refugees’ psychological wellbeing post-resettlement [3, 4]. Peri- and post-natal healthcare is one such service essential for many refugee women [5]. Globally, disparities in maternal health outcomes exist between women of refugee backgrounds and non-refugee backgrounds, including inequalities in maternal morbidity, preterm births and stillbirth rates [14–16]. Refugee status is also a known risk factor for peri- and post-natal mental ill-health [17].

Unfortunately, research suggests that the needs of refugee women in many resettlement peri- and post-natal healthcare settings are not always met due to challenges including understanding culturally relevant norms surrounding childbirth, and the impacts of psychological trauma [5, 14, 18, 19]. A small amount of research has been conducted nationally and internationally to address identified barriers to perinatal care and facilitate care that is more responsive to the needs of refugee women (e.g., [16, 18, 20, 21]). However, refugee focused perinatal services in Australia are limited, as well as comprehensive guidelines for best-practice peri- and post-natal healthcare for refugee women. Furthermore, few studies have examined the relationship between peri- and post-natal healthcare and psychological wellbeing among African-background refugee women, who may have unique needs due to experiences such as female genital mutilation (FGM) common in some African countries, as well as experiences of war, sexual assault, and life in refugee camps. In response to this gap, this project aimed to: (1) understand the relationship between psychological wellbeing and perinatal healthcare amongst refugee women from the African continent, and; (2) identify areas for improvement in perinatal healthcare in Australia in relation to the psychological wellbeing of this population.

## Method

### Study design

This study formed one component of a larger qualitative study concerning the maternal health needs of pregnant and post-natal women from African refugee backgrounds.

### Participants

A total of nineteen women with refugee backgrounds from African countries participated in the study. Two women were interviewed only whilst pregnant (third trimester), seven both while pregnant and after their baby was born, and ten only after their baby was born. Eligibility criteria included that women had arrived in Australia as refugees, had been in Australia for 10 years or less, were born in an African country, and were either pregnant or had a baby in Australia in the past year. Refugee women participants had arrived in Australia from nine different African countries: Sierra Leone (*n* = 5), Liberia (*n* = 4), Democratic Republic of Congo (*n* = 3), Somalia (*n* = 3), Ethiopia (*n* = 1), Burundi (*n* = 1), Ghana (*n* = 1), and Nigeria (*n* = 1)*.* All refugee women participants were aged between 19 and 43 (*M* = 29.45, *SD* = 1.99), and had lived in Australia from 11 months to 16 years (*M* = 9.71, *SD* = 4.92). Eight women had previously given birth in Australia, from as recently as 5 months and up to 13 years prior. (Six participants had also given birth to children in Africa prior to migrating to Australia, and of the seven participants who were pregnant, five had given birth to additional children previously, three in Africa.

Twenty service providers participants also participated in the study, including participants from refugee health services (*n* = 6), hospital settings including midwives and registrars (*n* = 9), and child and family services (*n* = 5). Participants had worked for between 6 months and 35 years (*M* = 12.44, *SD* = 2.57).

### Procedure

The Women’s and Children’s Hospital Human Research Ethics Committee approved the study (approval number HREC/19/WCHN/29) as well as the lead author’s institution. Refugee women participants were recruited with the assistance of African-background bi-cultural workers, while service providers were purposively recruited through the researchers’ networks and snowball sampling to cover staff from hospitals, maternal and child health services and refugee support organisations. Data collection took place between July 2019 and December 2020. After providing informed consent, participants took part in face-to-face and/or telephone or videocall interviews, with COVID-19 forcing all later interviews to be conducted via phone or videocall. All refugee women participants were offered the use of a professional interpreter, with two using a professional interpreter and two using family members.

The interview questions for refugee women explored women’s perinatal and general health backgrounds, health beliefs and norms around childbirth and care, experiences of perinatal care in Australia, impacts of those experiences on their psychological wellbeing, and their recommendations for improved care. For service providers, interviews covered their experiences providing maternity and post-natal care to refugee women from Africa, and their perception of the relationship between that care and wellbeing. Interview lengths ranged from 20 to 70 min (*M* = 37.87, *SD* = 11.83). Upon data saturation (with no new themes evident), interviews were ceased at nineteen refugee and twenty service provider participants [22].

Interviews were recorded and transcribed verbatim. As suggested by Tracy [23], an audit trail was maintained throughout interviewing, ensuring transparency and self-reflexivity by documenting notes on methodology and reflections on interviews.

The research team were all women, and included four academics from non-migrant backgrounds, a midwife from a non-migrant background working closely with refugee women, and two bicultural workers with African backgrounds and experiences of migration themselves. These bi-cultural advisors helped with study design, forming links between communities and the research team members undertaking the interviews, recruiting participants, data collection and analysis. Importantly, one of these advisors is an author on this paper and has a refugee background herself. The involvement of bi-cultural advisors was key to ensuring that interviews were conducted in a culturally safe and appropriate way, and importantly, the bi-cultural advisors indicated the preference of African background women to discuss sensitive topics such as maternity care, with someone far removed from their community. Moreover, the diversity of views from the research team meant that analysis and recommendations could be made in discussion and collaboratively, reflecting a range of views. More generally, power relationships between people – including academics—without refugee experiences exploring topics related to migration is an important consideration in the research process, and we explore this further in the Discussion.

### Data analysis

We used thematic analysis to analyse the data, following Braun and Clarke’s [24] approach of: (1) transcribing data; (2) familiarisation with the data; (3) coding and identifying dominant themes; (4) reviewing themes against the whole data set and producing a thematic map; (5) naming and defining themes, and; (6) finalising the analysis through writing. We coded the data both manually and using NVivo12; initially using a deductive approach to identify the data relevant to the research question (the relationship between perinatal care and psychological wellbeing), followed by an inductive, latent theme identification approach to explore participants’ perceptions regarding their maternal healthcare experiences. This approach then enabled us to form themes, as well as consider triangulation between participant groups [24, 25]. All authors cross-checked the resulting codes and themes, bringing their own positionality to this process to ensure diversity of both lived and professional experiences to this process of analysis.

## Results

Thematic Analysis of participants’ interview data identified four themes pertinent to the research questions: continuity of care and relationships with healthcare providers are crucial; culturally responsive care is very important; women want to be recognised as equal decision-makers in their perinatal care, and; negative perinatal healthcare experiences have long-lasting psychological implications.

### Continuity of care and relationships with healthcare providers

A key element of perinatal service provision identified by both groups of participants as important for wellbeing was the relationships refugee women had with their midwives and other health practitioners. However, few women received care from the same midwife through their pregnancy, and thus developing trusting ongoing relationships was difficult. One exception was Aminata, a first- time mother from Sierra Leone, who was interviewed in her third trimester and then again when her baby was 13 weeks old. Aminata provided an account of the trust that was developed over time with her midwife who undertook home visits pre- and post-birth. During pregnancy, she said:

*…so she listens, and when I was I think seven months, and then we started talking if I wanted to have a water birth or if I want to have anything given to me during labour, and she keeps asking me that over and over, just in case over the time I’ve changed my mind. So she’s pretty good with that, yeah. I trust her.*

Additionally, Aminata described her midwife building trust after the birth by behaving in culturally responsive ways, such as stepping back and allowing Aminata’s mother to assist with helping the baby to breastfeed, and not interfering with her cultural need to hold the baby for first three hours after birth. After she had birthed her baby, Aminata said:

*She’s very good communicating with her patients and she understands that every person, every pregnant person is different. So, she tries to meet your needs in the way that you feel comfortable… So I think for her, she treats everybody the way they’re meant to be treated, because she doesn’t treat everybody the same. She kind of - she understands what people’s individual needs are […] she’s done it all. She’s worked with a lot of African women and she understands that they have their own beliefs and way of doing things.*

On the other hand, Gloria (DRC, gave birth in Australia four months prior), recalled her experience of seeing different practitioners at almost every visit while pregnant, and indicated that “*increasing the visits of midwife”* after her baby was born, particularly from the same midwife would have been ideal. Indeed, more visits from a trusted midwife after pregnancy was particularly important for women who were socially isolated. For example, Zala (from Ethiopia), who had a caesarean section and was in Australia on her own without her husband, noted:

*The [care I received in] hospital is okay but when I come home it was very hard. Then they give me to take some - like some medicine. So, they give me two…The other one is very strong, the other one is okay. But they tell me if you very pain, you can take the strong one, but they say you have to make sure someone is with you […] sometimes they come my friends. Yeah but not every time so one day I was very pain and then when the baby sleep I take the tablet. Then, it just made me sleep. I can’t get up. The baby is crying. I can’t get up. Then next time I didn’t want to do it. I was scared that time. It’s too difficult if no one to look after you. It’s too difficult.*

Continuity of care—both during and after pregnancy – was also consistently identified by service provider participants as an element of health care needed by refugee women from African backgrounds to support wellbeing. They noted that a lack of continuity of care precluded a trusting relationship, without which women may find it difficult to disclose information pertinent to their care, such as a history of rape, trauma, or FGM. For example, Naomi, a refugee healthcare specialist, said:

*Each time you have to restart again. And you have to tell the same story, you have to... and yeah that kind of creates a kind of, yeah you don’t want to tell everything, because you need to, to get the relationship with your professional, and they don’t get it because you don’t see the same person each time. Each time you go there it’s someone different.*

Complementing continuity of care, both service providers and refugee women spoke about the use of bicultural social workers positively, as an option that helped women advocate for themselves, and therefore for more control over the health care they received. For example, a bicultural social worker provided reassurance and an additional layer of care in the case of Esi, a 19-year-old woman from Ghana who had given birth in Australia 18 months earlier. Esi was assigned an African background bi-cultural social worker during her second trimester which helped her manage her fears in relation to the high blood pressure and mental health issues she was experiencing during her pregnancy:

*Because on the first trimester one of the nurses had asked me if I went through depression and stuff, which I told them. So they provided me a social worker […] Yeah it was really great [….] She would come and visit me a lot, she would attend to my appointments […] It was really good because it had made me forgotten about a few things that I’d been worrying about.*

Overall, refugee women from African backgrounds discussed positive relationships with healthcare providers as a key component of wellbeing, by providing social support to women who may otherwise be isolated, and by building trust and rapport. Correspondingly, service providers saw continuity of care – including with bi-cultural workers—as a key mechanism for ensuring positive wellbeing outcomes for African background refugee women.

### Culturally and refugee responsive care

Refugee women participants reported experiencing a variety of culturally unsafe perinatal healthcare practices, particularly in relation to privacy regarding disclosure or care for women previously subjected to FGM. Konnima from Sierra Leone and mother to an 18-year-old and 14-month-old recalled:

*She [a midwife] asked me some question. She did ask me about my, how some women have a women circumcision in Africa. If I'm part of it, and ‘how did I feel?’. I think those question was personal, and I told her that, “I don’t feel comfortable of you asking me to answer those questions” … The other question that she asked me, she knows that African, they have all these domestic violence things. “Have I gone through anything that will cause damage to my having the baby?” I told her that, “I don’t need to talk to you about those things. I think I'm here for my health” … There, she just says that, “look, it’s part of the government need to know about you”, but Australia is a multicultural country. Everybody have their own culture. Whatever have happening with me, my own culture issue … It’s my privacy.*

Here, Konnima details how she saw personal questions about issues such as circumcision and domestic violence as outside the remit of midwives’ work, and as private. Therefore, a critical component of culturally safe care, is better understanding of what is appropriate to ask about, and how to ask about issues such as domestic violence and circumcision in a way that is responsive to cultural norms and values of pregnant women.

Service provider participants also noted that refugee-centred care was critical, including due to the likelihood that refugee women from African backgrounds had undergone FGM. Service provider participants similarly indicated that medical staff needed to be more sensitive and aware when discussing obstetric and gynaecological issues, especially in the context of FGM. Wendy (a refugee health specialist) discussed midwives being unprepared to encounter women who had undergone FGM, and the negative psychological implications for a woman when this occurs.

*Particularly for African women, not lumping everybody all together but making sure that there’s very sensitive history taking around FGM and proper kind of birth planning and support if that’s really different because my experience or what I know is that most midwives kind of run a mile rather than talk to women sensitively about history of FGM. And there’s been some incidences where you know it hasn’t really been approached very sensitively and there’s been no proper birth planning and history taking and staff have reacted very poorly in a clinical situation.*

Notably one of the refugee women participants – Brigid (Sierra Leone)—shared that she was able to advocate for a caesarean section due to experiencing FGM:

*Because back in Africa they did the – you know the thing where they cut your private parts – so I was scared that I could – I won’t [be] able to push the baby and I told them that I just wanted C-section. They respect my wish.*

Other participants, however, felt that, due to experiences of culturally unsafe care by healthcare practitioners, they would always be disadvantaged and receive suboptimal perinatal care. For example, Lisa (Liberia) commented that she felt like a *“guinea pig”* during her maternity care. Similarly, Ariana (Liberia), suffered a traumatic stillbirth experience in an Australian hospital six years prior to her interview, and felt that she was disadvantaged in her ability to gain information about what happened due to her background:

*Each time I think about it I promise myself to never go to that hospital because I was so scared of them. My family talk to me, ‘oh just leave it, you don’t know how to speak English. You are African. This is the hospital. It’s the government hospital. If you sue the government, you won’t get power’.*

Finally, some refugee women participants discussed inadequate care for women with acute and significant mental health issues during pregnancy, including suicidality. For example, while Brigid was able to advocate for her needs with regards to having a caesarean section, she also recounted significant experiences of trauma, and shared with the interviewers her multiple suicide attempts as a consequence. At the time of her first interview, she had given birth to three children in Australia, and all had been removed from her care. Speaking in relation to her pregnancy with her first child, when she was in a closed mental health ward at a public hospital in Australia, she said:

*I was detained because I was feeling suicidal. So I was detained until I had my daughter [name]. I was in… the closed ward. So I was until – I think I was there for three months until I had my daughter because they were worried for the safety of the baby and the safety of [me] … I didn’t like it there because mixing with other – other people who have – people that have their own mental health issues. I just felt like that wasn’t the kind of place for me. I’m pregnant. What if someone hit me. What if – you know? All those [fears].*

In this instance, Brigid recounted care that she did not feel was suitable for her needs in an extremely distressing situation. Brigid’s care in the closed mental health ward is a key example of care which is not responsive to the needs of refugee women, for whom detainment may have particular implications in relation to prior experiences of trauma, and in which the voices of refugee women – in this case Brigid – are not likely to be heard.

### Women as equal decision-makers in their perinatal care: The importance of consent and control

As seen in the previous theme, many refugee women felt that they were not adequately acknowledged or cared for during and after their pregnancy, and the ability to have input and some control over experiences was seen as particularly important. In the accounts of refugee women participants, there were few instances of women advocating for their needs and being heard. In one of these instances, Jernora (Liberia) had an emergency caesarian section and fought to see her baby, although not without initially experiencing significant distress when she thought her baby was dead:

*I never see my baby until the next day […] I don’t believe my baby was alive. In the morning they come told me they say Jernora it’s shower time. I said no, I want to see my baby first, see baby is alive. They say yes baby is alive. I say no, I will not take shower… until I see the baby. They took me there.*

In most cases, however, women recounted instances of feeling that they were not listened to nor able to have input or control over their experiences – in some cases resulting in feelings of neglect. For example, in addition to feeling that she was asked inappropriate questions, Konnima also indicated that she felt her care was perfunctory and that she was not listened to:

*I wasn’t feel like I was listened to … it’s just like when you go, they tell you, “hop on the bed, check that, and come down. Oh, the baby is fine”. That’s it. Sign you off, and you leave … So I feel neglected, actually.*

In addition to feeling neglected, some participants felt that their requests were ignored by medical staff. This was particularly the case in relation to the gender of the midwives attending to women, with most refugee women participants noting a strong preference for women, as would be culturally appropriate in most cases. However, these requests were often ignored, as Layla (Liberia) said:

*I did have a female and then they switched without telling me. Then when I came in, it was a male. Then I said no, I don’t want to see a male as a midwife. I want a female but even up until now, when I go, I still see male. So it’s basically, I personally feel like it’s just take whatever you get … that’s why now, even I was on Tuesday, I was supposed to go for one of my appointments … but I just felt like “no”. I didn’t go … I don’t feel like going and they even said they want to induce me this week.*

As seen in Layla’s account, ignoring women’s wishes about aspects of care such as women midwives could lead to disengagement from services, including not following advice about delivery. Service provider participants also expressed concern over the degree to which all available options were explained well in a clinical setting, so that women understood the nature of the care options they were given and the consequences of each option. For example, Joanne (a midwife) talked about a *“loss of control”* women may feel over decision-making during the perinatal period.

Refugee women – particularly those who had already had children – also discussed the importance of being recognised as knowledgeable about their own pregnancy and childbirth needs. However, most refugee women did not feel that they received this recognition, which in Ariana’s (Liberia) case was perceived to have catastrophic consequences. As noted above, one of Ariana’s babies had been stillborn in Australia, and she recounted the events leading up to this as follows:

*The midwife ring the department and told them that “oh the lady (.) the African lady is here, she been crying that oh she’s ready to give birth but water is not breaking. But now I have checked on her, the baby head is right there. She’s ready to give birth”. They [the medical staff] refuse … So they sent me home. When they send me home my baby pass away. Three days in my stomach … the day they [the medical staff] was ready for me to give birth the baby’s gone.*

For Ariana, the lack of control and recognition of her own knowledge about her body – as well as her African identity and the associated perceptions of staff—was seen to have a direct impact on the stillbirth of her baby.

Service providers concurred with the importance of ensuring refugee women were given some control over their care, and noted that where this wasn’t the case, feelings of anxiety and loss could develop, even if the pregnancy and birth ultimately went smoothly from a medical perspective:

*And the nature of course of maternity care is that things change very quickly and sometimes emergency decisions are made and that’s difficult for anybody, but extra difficult when you’re from that background where you can’t just access all things we can, like talking to somebody and asking your friends. So, I think it’s, it probably does impact on anxiety and maybe like lack of satisfaction about how things have gone. You know, maybe even some loss that they didn’t have that control over what they wanted to happen during the pregnancy. (Ellen, general practitioner)*

Another issue concerning control during maternity care specifically expressed by multiple participants was insufficient consent regarding the presence of student midwives. For example, Layla indicated that her request that no students be involved when she gave birth to her second child was ignored by hospital staff resulting in significant harm to her wellbeing, particularly given her past experiences and the unprofessional behaviour exhibited by some of the students:

*I did request as well was I didn’t want a whole group in the room. I didn’t want the students […] I was circumcised when I was young. I was forced to. Then I did get not rightly circumcised. This is very personal…. I felt like … I know that when somebody sees and you’re used to seeing clits and stuff like that, then when you don’t see it, you’re confused. Then with me as well, when I did get circumcised, I got cut wrongly so all that. I feel like when those students are in, they’re not experienced on facial expressions and stuff. So, it just made me feel a bit… I was in pain but then it’s funny how you’re in pain but your other side of the brain is also concentrating on that part. Like oh, they’re judging me… You shouldn’t care when you’re giving birth but yeah.*

As such, Layla recounted experiencing loss of control both in relation to the gender of the midwives that cared to her and in relation to the presence of student midwives.

However, other participants described positive experiences in relation to consent, which they found reassuring. For example, during the follow up interview, Bilan (Somalia) indicated that when she gave birth to her baby, the midwives were highly consultative in their care, giving her *“courage”*:

*When I’m in labour, yeah, they’re very, very, very helpful. They talk to me. They say we do the way you like it. They tell me, this is what we can help you if you like this way…. I’m very happy, yeah [they] give me courage. Courage, yeah.*

Bilan had previously indicated a high level of satisfaction with the care that she was receiving when interviewed while pregnant: “*I’m very satisfied with them and I feel like they give me a good support and I’m very comfortable with them, yeah.”* Similarly, Aminata—the only woman to receive continuity of care through a midwifery program—detailed the communicative and adaptive approach to care that her midwife took at all stages of her pregnancy and birth as seen above.

### Perinatal healthcare experiences have long-lasting wellbeing implications

Experiences with perinatal healthcare services in Australia – especially those which were negative—were found to have significant and enduring impacts on participants’ wellbeing. For some women, mental health challenges occurred as a result of traumatic birth experiences, such as Ariana’s experience of stillbirth described above:

Since then [the stillbirth of my baby] I’m not normal anymore. To be honest. I’m making myself stronger for my kids.

Like Ariana, other participants discussed psychological coping mechanisms in order to manage their own distressing experiences. Lydia (Burundi, gave birth in Australia eight months prior), reported suppressing difficult thoughts and memories of her birth in order to cope:

*My mental health I can say is good because I don’t feel anywhere pain … if something make me [feel] pain [mentally] I don’t think about that. I throw out … I don’t keep in my mind. Always put away because if I think about it I kill myself so put away.*

Here, Lydia indicates that while she has experienced negative events so serious that they make her contemplate suicide, she has ways to cope with the emotions these events elicit (namely, not thinking about it).

However, while some women such as Lydia said they had good coping mechanisms, it is noteworthy that for participants who had babies in Australia, negative experiences with perinatal healthcare services – and the extensive impacts on their wellbeing – catalysed two responses. For some women, the trauma and emotional impacts of their experience instilled determination to pursue better future outcomes, both for themselves and for other women, as in the case of Konnima:

*[My perinatal care experience] was very hurtful… I think, with this experience I have, if I get pregnant again, I’ll not close my mouth again, because I went through a lot.*

For other participants, the extent of their dissatisfaction with care created hesitation around seeking formal perinatal healthcare in the future. Underpinning this apprehension was a perception that perinatal healthcare of a high standard is ‘lucky’ to find for women from African backgrounds, as Layla explained:

*Because if you’re with the hospital in at [suburb removed] area … a lot of people would be like, “oh they’re so racist”. That’s how they will say to me … “they’re so bad, they don’t even care about you. They don’t”. Yeah, so they’re always telling me I’m lucky to be at the [hospital name removed].*

In addition, service provider participants acknowledged the role previous trauma had in the shaping of a woman’s pregnancy, childbirth, and parenting experiences. For example, Wendy (a midwife) and Naomi (a refugee health specialist bi-cultural worker) gave clear examples of the bidirectional relationship between trauma and maternity care. Naomi described incidences of women having flashbacks of previous traumatic experiences, while Wendy described the insensitivity of particular maternity practices such as internal examinations in situations such as when a woman has experienced rape. Again, participants called for woman-centred care that fosters sensitivity and understanding in health care professionals:

*Yeah sometimes like, women who have been raped or who saw whatever things in the war, when they are pregnant it can come back and lead to mental health ... They can have flashbacks of what they saw or they, what happened. It can lead to mental [illness] (Naomi).*

*But you know lying on your back with your legs open with a man standing over the top of you kind of talking at you, you know it’s a pretty violating experience for any woman let alone a woman who’s been held down and raped (Wendy).*

Further, service providers indicated that given the extent of mental illness experienced by many refugee women from African backgrounds, “*mental health care should just be a part of perinatal care”* (Ellen). However, care focused on wellbeing needed to be provided in a way that is culturally safe as well as sensitive to past experiences of trauma associated with being from a refugee background. This is particularly important because, as service provider Carla (a midwife) noted, mental health is often “*not something you discuss.”*

Overall, negative experiences with Australian perinatal healthcare services had significant and enduring impacts on the psychological wellbeing of refugee women from African backgrounds. This theme identifies how these experiences – which may compound previous trauma associated with the refugee experience – can shape the status of women’s mental health into the future and create apprehension towards utilising healthcare services.

## Discussion

The results of this study provide valuable insights into perinatal healthcare services in Australia and how they affect the wellbeing of refugee women from African backgrounds. In particular, the results highlighted how system-level factors and a sense of autonomy throughout care, all affect wellbeing outcomes amongst refugee women from African backgrounds post-resettlement. These results corroborate many of the findings of past literature, whilst also adding novel contributions to the evidence base in the Australian context, and key recommendations for improved perinatal healthcare for this population.

### Overview of findings in relation to previous literature

A key finding of this study was that perinatal healthcare system-level factors, particularly continuity of care, affected participants’ psychological wellbeing, an important finding with significant practice implications. Participants stated that how they were treated by medical staff was impactful, reflecting previous research [26, 27]. Equally, negative care experiences have been reported to cause notably poor wellbeing outcomes including apprehension amongst patients to disclose their feelings, wishes and needs; thereby placing women in a position of vulnerability [26].

Overall, continuity of care was perceived as best-practice perinatal care by both groups of participants. Previous research also notes the importance of continuity of care for relationship-building, detecting mental health and other issues such as family violence, improving wellbeing outcomes, increasing maternal health literacy, and providing reassurance for women in an unfamiliar healthcare system [26, 28, 29]. However, few participants in this study experienced care continuity. Participants who previously gave birth in Australia also discussed at-home postpartum follow-up care as important for refugee women who may otherwise be isolated, reflecting recommendations from previous research among other populations [29].

The need for improved culturally safe care [30] was also strongly emphasized, and was seen in some examples provided by participants such as cases where women were able to engage in cultural practices such as bonding with babies for extended periods immediately after birth. On the other hand, participants described encounters with medical staff who had limited awareness of how culture shapes pregnancy-related experiences and preferences. This was perceived by some participants to fuel negative assumptions about them and their fitness to parent. Misinterpretations amongst healthcare practitioners in relation to culturally diverse childbirth beliefs are not uncommon [31] and can lead to negative outcomes, including distress, as was experienced by participants in this study. Culturally unsafe care was seen particularly in relation to a lack of respect for participants’ privacy, including concerning culturally relevant topics such as FGM; also documented in other Australian and international studies [16, 18, 21, 26]. Participants’ experiences of perinatal care which failed to be culturally safe contributed to a belief that they would receive disadvantaged care as a consequence of their cultural background; and indeed, previous Australian studies have shown that culturally diverse women were less likely than Australian born women to have their maternity and post-natal care needs met [21, 32].

Importantly, discussions of culturally safe care also point to the need for trauma-informed practice. The tenets of trauma-informed practice as they are commonly understood—including ensuring patients’ safety, building trust and rapport, and empowering patients—are directly aligned with ensuring cultural safety more generally and thus working to embed trauma-informed practice in maternity care will enhance efforts at cultural safety, and vice versa. More generally, trauma-informed care can be seen to cut across all themes found in this study, including in relation to empowering women as equal decision-makers in their care and consideration of the ongoing impacts of perinatal care experiences. As such, trauma-informed practice is a key practice recommendation to stem from this study.

While some of the above suggestions could be applied to all culturally diverse women—particularly those from African backgrounds in the case of FGM especially—the findings of this study also pointed to the need for refugee-sensitive perinatal care which acknowledges the psychosocial challenges and implications of refugee status, including previous experiences of trauma. This supports other research [29, 33] and indicates that it is crucial for perinatal practitioners to understand the particular needs of refugee background women. There are very few refugee-specific perinatal services in Australia. Such services could include the provision of psycho-social support from trained bi-cultural mental health staff, as well as peer support groups of women from African backgrounds who are all at similar pre- or post-natal stages and therefore may be experiencing similar challenges and requiring similar support [21]. In these instances, facilitators or staff could be trained in the provision of culturally safe, trauma-informed care as discussed above, to ensure that refugee background women receive care that is targeted and helpful. Beyond refugee-specific services, refugee-sensitive care may also include elements that are easier to implement, such as training in issues that refugee women may have experienced to a greater degree than other women, such as the impact of sexual assault or FGM. In fact, the findings from this research project have fed directly into a partnership program between a key South Australian African Women’s organisation and a local hospital. This partnership seeks to bring together African women and hospital staff to facilitate such training and knowledge transfer. Notably, since some of the experiences identified in the research are not unique to refugee women, it is likely that such training will greatly benefit all women accessing perinatal services. Overall, greater knowledge and sensitivity amongst perinatal practitioners to ensure refugee-responsive care is essential for ensuring positive wellbeing outcomes in the perinatal period and beyond.

Another key finding from this study was participants’ wishes to be recognised as equal decision-makers with a degree of control. Some participants (many of whom had given birth previously) felt that they were provided with insufficient support, mirroring another Australian study with African-background women [27]. Other participants felt that they were not treated as knowledgeable or autonomous in their perinatal care and were not listened to by hospital staff. For some women this may be especially important as pregnancy and childbirth can incite feelings of empowerment and control [30]. There is a known relationship between feeling in control during the perinatal period and positive wellbeing outcomes; where validating women as experts and elevating their strength as mothers can also contribute to achieving positive postpartum health goals more generally [34, 35]. However, many participants in this study felt powerless, which was contributed to by inadequate consent processes regarding involvement of student midwives in particular. Little research has investigated this specific issue, though some research has explored similar issues regarding medical students more generally [36]. Overall, supporting women to feel valued and recognised as key decision-makers throughout their perinatal care is essential for promoting positive wellbeing outcomes, and for ensuring continued service use.

Finally, participants’ discussions of the long-lasting mental health impacts of perinatal healthcare experiences highlighted the significant implications for psychological wellbeing. Multiple participants were deeply negatively affected emotionally by their experiences of perinatal care, with feelings of regret and anger, and sometimes suicidal ideation. Suicide is a leading cause of maternal death in developed countries, making it essential that women receive quality perinatal healthcare [37]. For many participants, the psychological turmoil following their perinatal care experiences instilled apprehension towards seeking support in future; a result also found by Mannaya, et al. [4]. It is crucial that women are supported to seek perinatal care given the existing relationship between low service use and mental health challenges in pregnant women [37]. In this study, participants described a community consensus that good quality perinatal healthcare was ‘lucky’ to find for African background women, likely reducing service engagement and fueling already pervasive health inequalities amongst refugees in the post-resettlement context [26].

### Study limitations and future research

While the study made an important contribution to knowledge in this area, it is not without its limitations. In particular, it is important to acknowledge the potential limitations of the coverage of experiences of participants involved in this research. Although every effort was made to include the refugee women experiencing the greatest vulnerability, some may not have had access or means to participate, meaning the participants may represent those with particular resources or interests. It is also worth noting that the later stages of recruitment and interviewing intersected with COVID-19 restrictions, creating challenges for recruitment (e.g. due to social distancing policies, fear, or lack of resources) and participation (e.g. the technical and rapport-building challenges of interviewing via telephone).

Additionally, the scope of this study – focusing specifically on the experiences of women with refugee status – addresses limitations of the current evidence base where those with refugee and migrant status are often combined under a singular definition. Exploring deeper insights into the perspectives of women of refugee backgrounds specifically offers recognition of the uniqueness of their experiences and contributes to specific recommendations for improving psychological wellbeing outcomes in the perinatal context. The findings of this study lay the foundations for future research, including deeper exploration of cultural differences amongst women from different African countries and cultures. Further research with participants of asylum seeker backgrounds would also be valuable, as those with asylum seeking status are generally less supported by healthcare systems in resettlement countries, and therefore may face additionally unique challenges in the perinatal healthcare context.

Finally, as noted in the methodology above, we reiterate that the composition of the research team disproportionately included non-migrant women and academics. We tried to account for this, and the associated power differentials, in the design, conduct, analysis, and reporting of this project, and it is worth noting that the project involved extension collaboration and co-authorship with women of African background who had refugee experiences. Nevertheless, these power differentials should be considered in relation to the research findings.

### Conclusion and recommendations

With a dearth of literature on this topic and few refugee-focused perinatal services currently operating in Australia, nor comprehensive guidelines for best-practice perinatal care for refugee women, this study addresses a crucial literature gap regarding how experiences with mainstream perinatal healthcare services affects the wellbeing of African-background women with refugee backgrounds. In particular, change is required at the system level to promote the successful implementation of practice recommendations, highlighted by the findings in this study. Firstly, employment of a continuity of care model is recommended to ensure a high standard of individualised perinatal care, which also recognises the role of patients in informed decision-making. Secondly, it is crucial that practitioners are educated and competent in providing culturally- *and* refugee-competent perinatal care to patients that empowers women to be active agents in their care. Finally, Australian perinatal healthcare services must be equipped to provide refugee women from the African continent with tailored and trauma-informed support. This support must recognise the potential psychological impacts (and the extent of those impacts) of perinatal healthcare service experiences on women’s wellbeing. Providing optimal care to women from refugee backgrounds at this critical time of life is crucial for improving wellbeing outcomes for this group of women.

## Data Availability

The datasets generated and/or analysed during the current study are not publicly available, as participants in the study did not provide consent for their data to be shared with other researchers. Questions about the data can be sent to clemence.due@adelaide.edu.au.
